# Population coding of figure and ground in natural image patches by V4 neurons

**DOI:** 10.1371/journal.pone.0235128

**Published:** 2020-06-26

**Authors:** Yukako Yamane, Atsushi Kodama, Motofumi Shishikura, Kouji Kimura, Hiroshi Tamura, Ko Sakai

**Affiliations:** 1 Graduate School of Frontiers Biosciences, Osaka University, Osaka, Japan; 2 Japan Society for Promotion of Science, Tokyo, Japan; 3 Department of Computer Science, University of Tsukuba, Ibaraki, Japan; 4 Center for Information and Neural Networks, Osaka, Japan; Johns Hopkins University, UNITED STATES

## Abstract

Segmentation of a natural scene into objects and background is a fundamental but challenging task for recognizing objects. Investigating intermediate-level visual cortical areas with a focus on local information is a crucial step towards understanding the formation of the cortical representations of figure and ground. We examined the activity of a population of macaque V4 neurons during the presentation of natural image patches and their respective variations. The natural image patches were optimized to exclude the influence of global context but included various characteristics of local stimulus. Around one fourth of the patch-responsive V4 neurons exhibited significant modulation of firing activity that was dependent on the positional relation between the figural region of the stimulus and the classical receptive field of the neuron. However, the individual neurons showed low consistency in figure-ground modulation across a variety of image patches (55–62%), indicating that individual neurons were capable of correctly signaling figure and ground only for a limited number of stimuli. We examined whether integration of the activity of multiple neurons enabled higher consistency across a variety of natural patches by training a support vector machine to classify figure and ground of the stimuli from the population firing activity. The integration of the activity of a few tens of neurons yielded discrimination accuracy much greater than that of single neurons (up to 85%), suggesting a crucial role of population coding for figure-ground discrimination in natural images.

## Introduction

**Fig 1:**
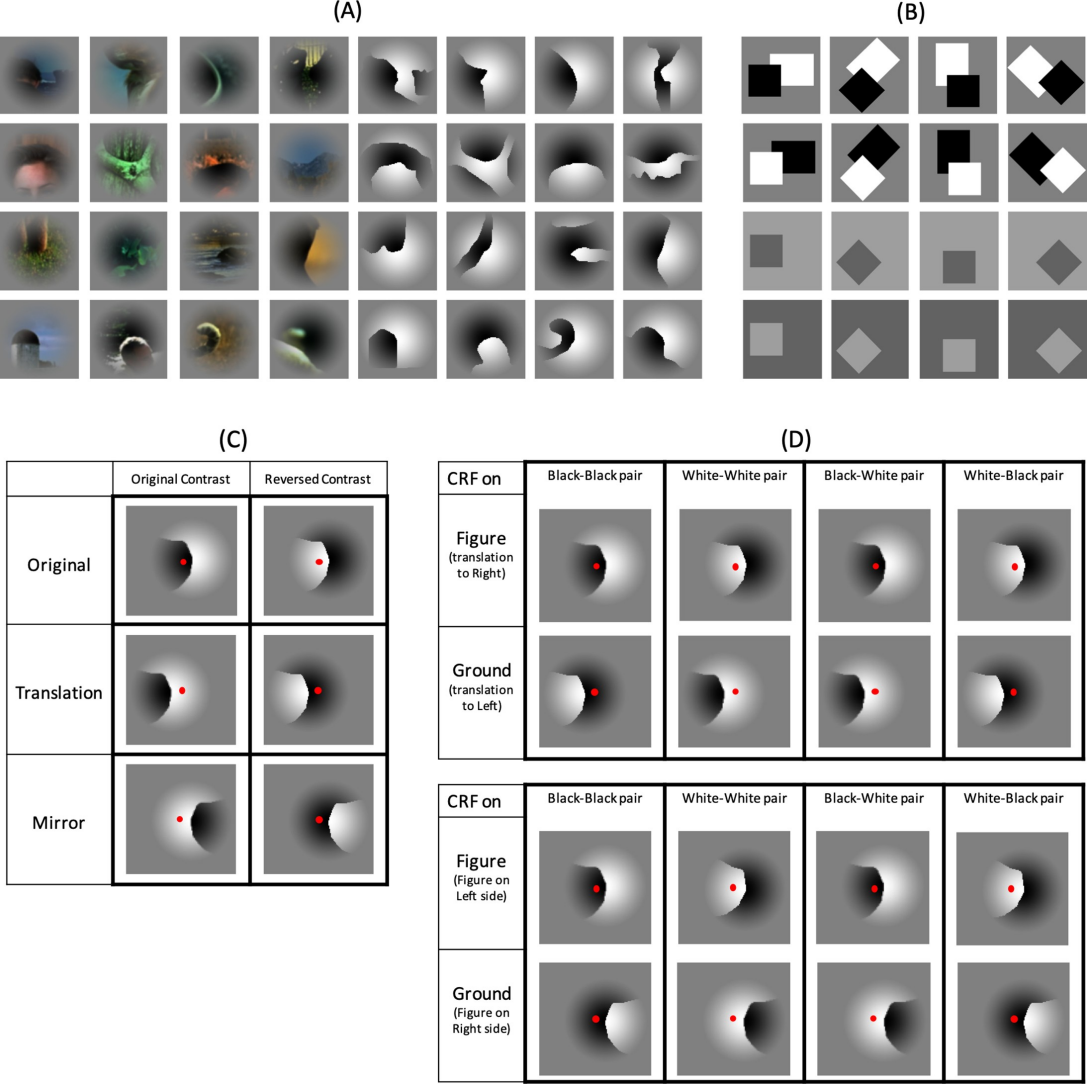
Some examples of stimulus patches taken from the Berkeley Segmentation Dataset. A: Examples of original natural patches (A, left). In the present experiments, 105 of these patches were used. Filled patches (A, right) were created by retaining contours and filling the rest of the space with black and white. B: Examples of conventional square stimuli with contrast and orientation variations. C: Schematics of example relations between image patches of different conditions and a CRF center. Red dots indicate the CRF center position, which is the same across conditions. Note that the CRF center is included for presentation purposes and was not shown in a real stimulus. The translation pairs (TPs) evoke responses to figures and grounds if the CRF center is located relatively close to the patch center, while the mirror pairs (MPs) evoke such responses if the CRF is located further away. D: The combination of luminance contrast and FG. The upper set (TP session) includes TPs, while the lower set (MP session) includes MPs. The “Black-Black pair” indicates that a black figure fell onto the CRF in one patch, and a black ground fell onto the CRF center in the paired patch. For each pair, there were four contrast variations; black-black, white-white, black-white, and white-black. CRF, classical receptive field.

**Fig 2:**
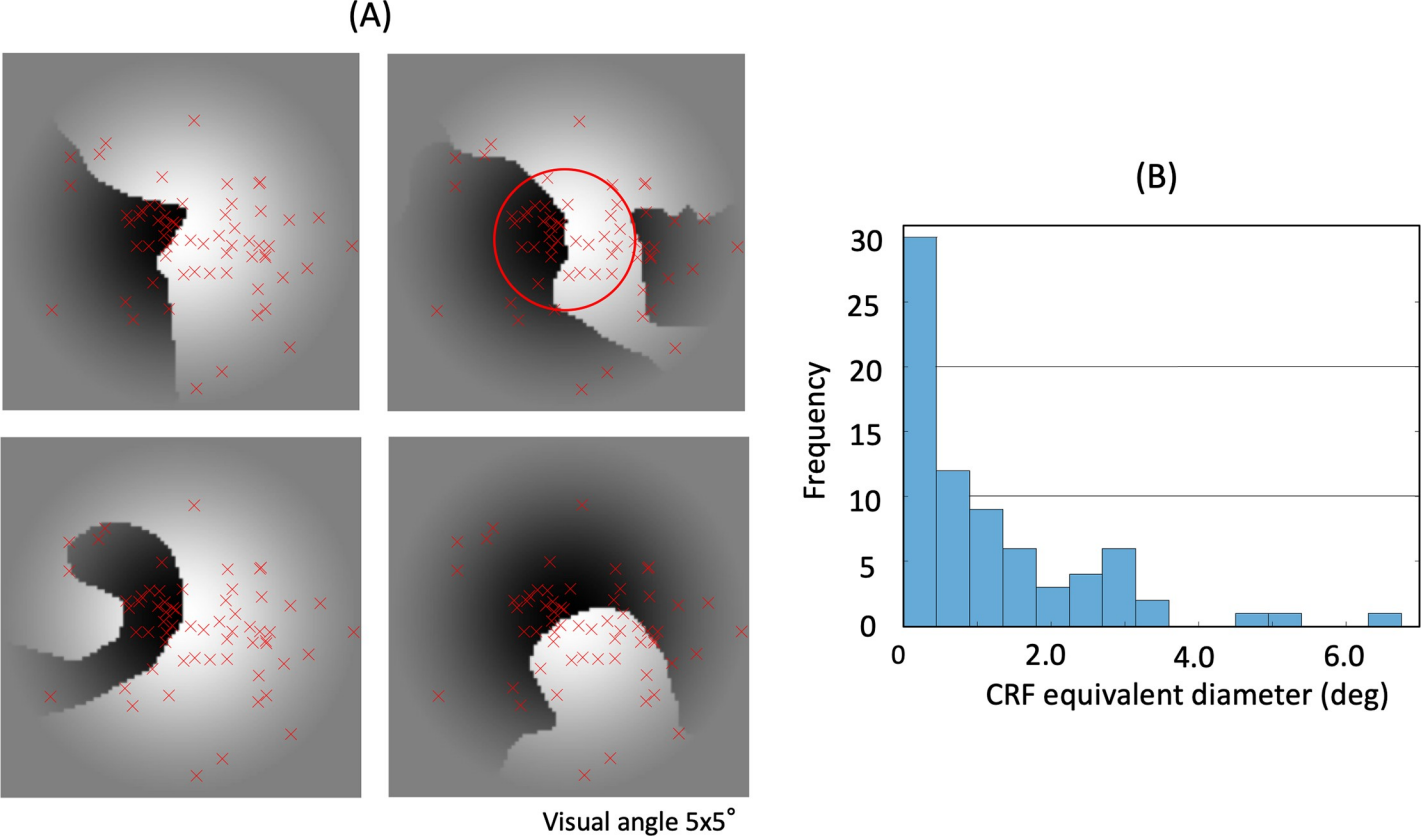
The distributions of CRF center positions and size of recorded neurons for an example single recording session. Each CRF was estimated by fitting a 2D Gaussian to the neural responses. A: The distribution of estimated CRF centers of neurons in an example recording session. CRF centers (red crosses) are overlaid on four example stimulus patches. The red circle at the center illustrates the mean extent of estimated CRFs (1.8 degrees in diameter). B: The histogram of estimated CRF extents of the neurons in the same session. The extent was defined as the diameter of a circle whose area is identical to the area surrounded by the half-height of the 2D Gaussian. The mean estimated diameter was 1.4 degrees in this session.

Segregation of images into figures and background is crucial for understanding scenes and recognizing objects. Physiological studies have shown a variety of neural modulations relevant to the organization of figure and ground in the low- and intermediate-level visual areas. A majority of V1 neurons exhibit significantly stronger responses to texture elements belonging to a square-shaped figure [1, 2]. Recent studies on texture segregation have suggested distinct processes for the enhancement of figures and suppression of background in V1 and V4 [3, 4]. A majority of V2 neurons have been shown to change their firing rates depending on the side of the figure or the border ownership [5]. In the detection of contours, V4 neurons were reported to exhibit enhanced responses to line segments that form a global contour, and suppressed responses to background line segments [6]. Investigations on shape coding in V4 have reported neuronal selectivity to curvature along a closed shape and a figural surface [7–9]. In all these pioneering studies, the responses of single cells to well-controlled stimuli, such as textured squares, luminance-defined rectangles, and parameterized curvatures, were examined with the careful selection of cells based on their stimulus selectivity (e.g., orientation, color, and shape) and the spatial relations of their classical receptive fields (CRFs) with respect to the stimulus. [10]

Recent studies have investigated FG organization in natural scenes, reporting crucial factors for FG based on local image features including contour shapes [11–13] and spectral anisotropy [14]. V2 neurons were shown to be selective to border ownership in natural images [15]. However, how neurons determine figure-ground (FG) from natural scenes has not been clarified yet. Investigations on population neural activity in response to natural images, rather than the responses of specific single-neurons to specific artificial images, are likely crucial to understand FG coding across a wide variety of FG organization and leads to the understanding of neural mechanisms underlying FG segregation in everyday natural scenes. Focusing on local information is also likely crucial in studying the construction of FG representation from natural scenes. Global context providing information such as surroundedness and objectness are effective for the segregation of figure; however, a variety of local geometric properties, including convexity and symmetry, also constitute the classic configural properties involved in FG organization [16, 17]. Natural images include rich local information, such as color, texture, and contour segments, which play crucial roles in FG segregation. Human observers are typically able to segregate figure and ground in local natural image patches that lack global information [13, 18]. Computational models based on surround modulation that pool only local information have been found to exhibit the ability to determine border-ownership [19, 20].

In the present study, we investigated whether a group of neurons could decode local information contained in natural images, and represent FG information in a distributed manner even in the absence of global context. We recorded spiking activities of a population of macaque V4 neurons, and intentionally included as many neurons as possible, without excluding neurons based on response rate or stimulus selectivity, in subsequent analyses to examine the ability of FG segregation in a neural population. A variety of natural image patches, which were carefully designed to preserve natural image characteristics without biases, and their variants, were presented as visual stimuli. First, we examined whether the activity of single neurons depend on the local FG configuration of stimulus images, and showed that approximately one fourth of the neurons exhibited significant FG modulation. However, the individual neurons showed low consistency in FG modulation across natural image patches (55–62%). Next, we examined whether a population of V4 neurons represents the local FG information that is effective for a wide variety of natural images. Specifically, we trained support vector machine (SVM) [21] and examined whether the integration of the activity of multiple neurons enables greater consistency in FG signaling across a variety of natural image patches and how many neurons are necessary to represent FG across the patches. The result showed that integration of the activity of a few tens of neurons yielded discrimination accuracy that is far greater than that of single neurons (up to 85%), suggesting a distributed representation of FG information in V4. These results suggest a crucial role of population coding for FG discrimination in natural images.

## Materials and methods

We recorded neural activities of three hemispheres of two female macaque monkeys (*Macaca fuscata*; body weight 7.1 and 5.2 kg) provided by the National BioResource Project (MEXT, Japan; http://www.nbrp.jp). All animal experiments were performed in accordance with the guidelines of the National Institute of Health (1996) and the Japan Neuroscience Society, and were approved by the Osaka University Animal Experiment Committee (certification no: FBS-13-003). The animals were housed individually in animal rooms. The individual cages were placed to allow the visual, olfactory, and auditory interactions with other animals in the same room. The interactions between the animals were carefully monitored to avoid the stress caused by dominant individual. The balanced cubed diet, fresh water, and fresh vegetables or fruits, environmental enrichment were provided in accordance with NIH guidelines. The health conditions of the animals, and their food and water intake were checked and recorded everyday by a professional caretaker. During the surgery, the animal was anesthetized by using the general method, and vital signals were monitored (details of the anesthesia and vital monitoring are described in below). To confirm recording positions, animals were sacrificed after all recordings by deeply anesthetizing them with an overdose of pentobarbital sodium and perfusion through the heart.

### Animal preparation

General procedures were similar to those described previously [22]. A head restraint device was fixed on the top of the exposed skull, and then the exposed area encompassing the recording area was covered with acrylic resin. Surgery was performed under full anesthesia by inhalation of 1–3% isoflurane (Forane, Abbott Japan, Osaka, Japan) in nitrous oxide (70% N_2_O, 30% O_2_) through an intratracheal cannula. The electrocardiogram, blood pressure, end-tidal CO_2_, and arterial oxygen saturation were continuously monitored with a patient monitor during surgery. An antibiotic (Pentcilin, Toyama Chemical, Tokyo, Japan) and an anti-inflammatory and analgesic agent (Voltaren, Novartis, Tokyo, Japan or Ketoprofen, Nissin Pharmaceutical, Yamagata, Japan) were administrated immediately after the surgery, which was continued during the first postoperative week. Approximately 1–2 weeks later, the monkeys were fully anesthetized again, to examine corneal curvature and optical power, enabling the selection of appropriate contact lenses so that images presented 57 cm from the cornea were focused on the retina. Photographs of the retinal fundus were taken and used as a guide to determine the position of the fovea at the time of stimulus presentation.

### Recording of neural activity

As a preparation for neural recordings, the monkeys were head-fixed and anesthetized by inhalation of 1–3% isoflurane in nitrous oxide (70% N_2_O, 30% O_2_) through an intra-tracheal cannula, then infused with the opioid fentanyl citrate (Fentanest, Daiichi Sankyo, Tokyo, Japan; 0.035 mg/kg/h) in lactated Ringer’s solution. We drilled a small hole (~5 mm) in the skull and made a small slit (2 mm) in the dura for electrode insertion. A solution of 0.5% tropicamide/0.5% phenylephrine hydrochloride (Mydrin-P, Santen, Osaka, Japan) was applied to the monkeys’ eyes to dilate the pupils and relax their lenses. The cornea of the contralateral eye of the recording hemisphere was covered using a contact lens with an artificial pupil (diameter, 3 mm). During the recording sessions, vecuronium bromide (Masculax, MSD, Tokyo, Japan; 0.06 mg/kg/h) was added to the infusion solution to prevent eye movements.

For recordings of the neural activity of V4 neurons, we used 32-channel silicon probes, arranged linearly (A1X32-10 mm 50–413, A1X32-10 mm 100–413), or probes with eight shafts (A8X1 tetrode-2 mm 200–312) (Neuronexus Technologies, Ann Arbor, MI, USA). There were no noticeable differences in quality of neural recording, i.e., the number of recorded neurons and the mean firing rate, between the probes (refer S1 for details). Collected neural signals were amplified (×1000) and filtered (0.5‒8 kHz), using commercial amplifiers and filters (Plexon Inc., Dallas, TX, USA), then sent to a computer controlled by a custom-made data acquisition program (MATLAB data acquisition toolbox, Mathworks, Natick, MA, USA) at a sampling rate of 20 kHz. Some of the signals were bifurcated and fed to a band-pass filter and a window discriminator for the purpose of hand plotting and CRF mapping during the experiment (refer to “Experimental design” section). For the main analyses, we sorted single-unit spiking activities off-line for each session. The details of the spike sorting are described elsewhere [22–24]. After the experiment, the recording sites were closed, then the monkeys received analgesics and antibiotics, and then they were returned to their home cages. Each recording session lasted up to 7 h and was followed by at least one week of recovery.

### Visual stimuli

#### Grating patches for CRF plots

For the estimation of the CRF center and size, we used square-wave grating patches with 12 variations of frequency (3) and orientation (4). Grating patches (0.5 or 1 degree in diameter) were shown in distinct positions (in a 5 × 5 grid, covering 3 to 12 degrees of visual angle). The total number of grating patch was 300, and each patch was shown 10 times in pseudo-random order in each session.

#### Natural image patches

Natural image contours were drawn from the Human Marked Contours (HMC) available in the Berkeley Segmentation Dataset (https://www2.eecs.berkeley.edu/Research/Projects/CS/vision /grouping/fg/) [11]. We selected 105 sub-regions (69 × 69 pixels) from the HMC that included the contours passing through the center of the patches. As the distribution of contour curvatures is highly non-uniform in natural scenes, we controlled the distributions of the degree of convexity, closure, and symmetry of contours (uniformly selected from each range of these characteristics) [13]. Several examples are shown in Fig 1A and all the patches are shown in S2 Fig. We prepared a variation that was the mirror image with respect to the tangent of the border passing through the patch center. The color of the mirror images was inverted so that the polarity of the color contrast remained constant with respect to the central border. Including a mirror-image variation of each patch, the total number of patches of this set was 210. Although the patches are a small part of a full image, they could contain contextual information, such as a half of a human face or half of a silo, as shown in Fig 1A. We did not exclude these patches from the stimulus set because no objective criterion for context was established. Not limited to the cases where a substantial portion of an object is visible, viewers could potentially infer what objects are present based on other information, such as texture, color, partial contour, or a combination of these features. Although patches with visible contextual information were included in the stimulus set, there were only a few of them (refer to “Discussion” section for the effects of the contextual information). Since the extent of the patches that corresponds to visual angle was approximately one tenth of the original full images, the probability of appearance of context was much smaller in the patches compared to full images. The standard deviation of the spatial distribution of luminance over all-natural image patches was 7% of the mean luminance, indicating that the average patch was close to a uniform mid-grey. To obscure the boundary between the patch and the grey background, we attenuated the contrast towards the periphery with a Gaussian function. We performed a psychophysical experiment to obtain the veridical FG labels for all patches [13, 25]. The experimental procedures and results are described in S1 Appendix. Perceptual judgments of figure and ground in these natural patches were not consistent across participants and trials, as reported in previous studies [11]. The mean perceptual consistency across all natural-patches was 0.69, with a standard deviation of 0.11 (see details for S1 Appendix). The consistency with the original and mirrored (color-inverted) patches was 0.78 and 0.60, respectively, indicating worse consistency with color-inverted images. The results indicate a fairly-wide variety of perceptual consistency in FG judgment across the natural patches.

#### Filled patches

The natural image patches described above were filled with black on one side and white on the other side. Examples are shown in Fig 1A and all the patches are shown in S2 Fig. In addition to the mirror pairs (MPs), we prepared a variation that was slightly translated orthogonally with respect to the figure-ground border, with the aim of obtaining responses to the figure and ground of each patch. The distance of translation ranged from 0.6 to 4.8 degrees depending on the scaling of the stimulus presentation (refer to “Experimental design” section). Including variations in contrast (2), mirror image (2), and translation (2), the total number of stimuli of this set was 840. Examples of the mirror pair (MP) and translation pair (TP) and the contrast combinations are shown in Fig 1C and 1D, respectively. The standard deviations of the spatial distribution of luminance over the original image patches and the set of TPs were both 5% of the mean luminance, and that over the set of MPs was zero, indicating that the average patch over the filled patches was close to a uniform mid-grey. We performed a psychophysical experiment to obtain the veridical FG labels for all patches. The experimental procedures and the results are described in S1 Appendix. The mean perceptual consistency across all filled patches was 0.77 with the standard deviation of 0.14, indicating fairly solid judgements in FG determination, to a similar but of slightly higher degree as compared to that across the natural patches (see details for S1 Appendix).

#### Square stimuli

Single squares and pairs of overlapping squares were also presented, similar to those used in previous studies on border ownership [5, 26]. Including variations in single/two (2), contrast (2), translation (2), mirror (2), and rotation (4), the total number of stimuli in this set was 64. A few examples are shown in Fig 1B.

### Experimental design

After inserting the electrode into the recording area, we isolated multiunit spiking activities from the signal of one of the probes (situated at the center of the linear array or the center shaft) using the window discriminator. We manually plotted the CRF center and checked the preferred color (if any); characteristics of the CRFs were then estimated by running a pre-examination session while presenting the grating patch pattern in different positions (refer to “Visual stimuli” section). The stimulus presentation positions were determined using the results of this pre-examination session. The color of the filled and square stimuli for the main experiment was also determined from the pre-examination, whereas the color of the natural patches was not changed. Stimuli were also scaled to cover the CRFs of the recording units, more than three times larger than the rough estimate of the CRF diameter, yielding a stimulus size between 2.5 and 21 degrees. We selected this scale to ascertain the recording from multiple neurons with their CRFs covering different regions of a stimulus with reasonable overlap. Stimuli were shown against a plain grey background on a 27-inch LCD monitor (CG275W Eizo; refresh rate, 60 Hz; white luminance, 125 cd/m^2^; black luminance: 1.3 cd/m^2^) placed at a distance of 57-cm from the monkey’s eye. All stimulus presentations were repeated 10 times within a session, in a pseudo-random order, and shown for 200 ms with a blank 200-ms inter stimulus interval. We separated the recordings into two sessions: TP and MP sessions which included the translation and mirror patches, respectively, together with the contrast-reversed patches (Fig 1D), the square stimuli (Fig 1B), and the grating patches. The grating patches were included to determine the CRF center and the extent of individual neurons. Natural patches were included only in MP sessions. Based on the inspection of the receptive field properties of each neuron (the center position and extent of the CRF and preference to color) the neural populations of recorded neurons appeared to differ between sessions.

### Data analysis

For the examination of responsiveness to stimuli, we compared the firing rates of isolated single units during the pre-stimulus period (-50‒+50 ms after stimulus onset) with those of the stimulus period (50‒250 ms after stimulus onset), for all stimuli, with t-tests. A value of p < 0.05 was used as the criterion for responsiveness.

To estimate the retinotopic location and extent of CRFs, we counted the number of spiking events in isolated single units during the presentations of the grating patches shown at different retinal positions. The center and extent of the CRFs were estimated from the mean spike count maps fitted by a two-dimensional Gaussian function. Based on the positional relation between the CRF center and the content of the image patch, we classified the patches for each neuron into four categories: CRF center on the figure or on the ground (FG; “figure” or “ground”), and on the black or the white region (contrast; “black” or “white”). These labels were used for the characterization of single units, the examination of response consistency, and the SVM analyses.

To examine the modulation of each neuron by FG and contrast polarity, we performed a two-way repeated-measures analysis of variance (ANOVA) on the responses to all filled and square patches (factorial design: response = FG + contrast + FG * contrast + noise). In the case of natural patches, we performed a one-way repeated-measures ANOVA with FG as a factor, because we did not have contrast-reversed stimuli for the natural images. The criterion for significance was set to p < 0.05. To clarify the dependence of the analysis on the distribution of data, we transformed the spike counts using the Anscombe transform, which approximately converts Poisson data to Gaussian distributions, and performed the ANOVA. The difference in the number of FG and/or contrast significant neurons was two (2/793 = 0.25%) between the original and transformed cases, and thus we did not apply the Anscombe transform for the analyses. The FG modulation for the *favorite* stimuli, rather than all the stimuli, was analyzed in a similar way. We defined stimuli as favorite if the response to the stimulus was greater than half of the peak response across all trials of the neuron. We also examined FG modulation based on the difference between firing counts corresponding to either paired-translation or mirror images. In some cases, the original and mirror/translated patches shared the same FG (e.g., the CRF of the neuron fell onto the figural region in both the original and translated patches). Such responses were excluded from the analysis. We performed an ANOVA on the difference of the responses, similar to the analysis performed for unpaired responses.

To assess the response consistency of each neuron, we obtained the correct rate of FG judgments (i.e., the number of correctly discriminated patches divided by the total number of presented patches). To judge whether discrimination was correct, we determined the threshold for FG judgments for each neuron. For instance, when a preferred stimulus was presented, we considered it correct if the response was greater than the threshold, but incorrect if the response was smaller. We used SVM [21] to determine the threshold. A choice-probability analysis [27] is a common method for determining the threshold from conventional single-cell-recording data. The present data were sparse as they were recorded in response to a variety of stimuli taken from natural images; therefore, we used discrete distribution rather than continuous probability. The simplest method suitable for the discrete distribution of sparse data would be a thorough search in which thresholds were placed at all levels of responses and the corresponding correct rates were computed. The greatest correct rate and the corresponding threshold could be used for the consistency analysis. Although a thorough search is perfect for the given data, this method lacks generality; there is no reason that this threshold works for other stimuli. To introduce generality for the consistency analysis, we used SVM, a standard classification algorithm known for its power in generalization [28]. By using a thorough search, the threshold is given between the two responses recorded in the session. For example, if the two responses were two and five spikes, then the threshold could be three or four spikes, but the method cannot tell which is better. Intuitively, SVM achieves a good separation so that determines which of three or four is better. The correct rates computed from the thresholds given by the thorough search and SVM showed a marginal difference in the present data (the mean difference between the two correct rates across the neurons was 0.25%). Moreover, the introduction of SVM is advantageous for the coherent comparison with the population analysis that used SVM. The preferred side (figure or ground) of each neuron was determined as the side with the higher mean firing rate. Neurons with fewer responses (spikes were observed in < 80 trials) to either figures or grounds were not taken into account to the analysis, in order to prevent classification error due to a small number of responses. This criterion excluded 47 of 193 FG-significant cells from the analysis. The details of the SVM analysis are presented in the next section (“Support vector machine”).

Onset latencies were calculated using the method described by Sugihara *et al*. [29]. The firing counts of all units under examination were plotted as a cumulative spike count histogram. The responses between -50 and 50 ms after stimulus onset were subtracted as baseline, and a moving average with a 5 ms window was applied. The two-phase regression fitted a pair of straight lines by minimizing the total sum of squared deviations. The intersection of the lines, which indicated the time point at which the firing rate changes, was taken as the latency estimate. For the FG-modulation latency, the two-phase regression was performed on a cumulative differential spike count histogram.

### Support vector machine

To examine whether the activities of multiple neurons were capable of signaling figures and grounds, we trained a SVM, an established classifier, to discriminate figures and grounds of the stimuli from the firing activity of neurons. We used Soft Margin SVM with Radial Basis Kernels in the LIBSVM library (https://www.csie.ntu.edu.tw/~cjlin/libsvm/). Free parameters [30] were chosen automatically using the Grid Search Method implemented in the library. Learning and testing of the machine were carried out with five-fold cross validation (80% and 20% of the data were used exclusively for learning and testing, respectively). Learning with 80% of the data was continued until the correct rate of classification reached 99%. The correct rate for FG classification was defined as similar to that of single-cell analysis: the number of correctly classified patches divided by the total number of patches examined. Upon successful learning, the machine was tested with the rest of the data. The partitioning of data into the learning and testing was random.

A dataset for input to the SVM was generated for each neuron. First, the sum of spike counts over 10 repeated trials for each patch (identified by image, natural/filled, contrast, and mirrored/translated) and its veridical label of FG (whether the figure or ground was projected onto the CRF center of the cell) were paired. The FG labels, either figure or ground, for the regions of the patches, were provided by human psychophysical experiments (refer to “Visual stimuli*”* section). The FG label at the location of the CRF center of a neuron was the veridical label for the combination of the patch and the neuron. Because the location of the CRF centers differs across neurons, different veridical labels could be assigned to the same stimulus across neurons. Next, the same number of patches with the veridical label of figure and that of ground were sampled randomly without repetition to generate the dataset. It should be noted that, depending on the combination of a patch and a CRF location, the numbers of patches with figure label and ground label would be expected to vary among neurons. For example, this was often the case when the CRF was far from the center in the direction of the tangent to the contour at the center, so that many recorded responses corresponded to grounds but few corresponded to figures. To equalize the contribution of figure and ground, we sampled the same number of patches with figure and ground labels. The dataset for each neuron was comprised of the list of the spike count and veridical label, but it did not include the identification of patch. Intuitively speaking, the SVM would be learned to predict the label from the spike count.

The pre-determined number (one, multiples of ten, *etc*.) of neurons were randomly sampled without repetition across multiple recording sessions, and their dataset were input to the SVM with their cell identification attached. The number of neurons to input corresponds to the dimensionality of the classification problem. For instance, if the integration of the responses from two neurons was examined, the problem was considered as searching the best boundary between the figure and ground in the two-dimensional (2D) space with each axis corresponding to the response of each neuron. By testing a range of numbers of input neurons, we examined how many neurons should be integrated to achieve a reasonable level of classification. The input neurons were interchangeably referred to as integrated neurons. Neurons with fewer responses (spikes were observed in < = 80 patches) to either figures or grounds were not taken into account, in order to prevent classification error due to a small number of responses. Among the FG-significant cells, this criterion excluded 15 of 193 (8%) neurons from the analysis (92% were "valid"). In the analysis of FG-significant cells, the preference to FG was ignored in sampling, so that they were randomly mixed. With natural and paired-translation patches, the number of valid neurons for integration was limited to 29 and 36, respectively. A learning and test set for each condition was repeated 200 times with a random selection of neurons and patches, and the results were averaged over.

## Results

### Local information in natural image patches and their relation to figure and ground

We presented image patches from natural scenes so that only local information was available, assuming that these patches included inherent cues usable by neurons or networks for inferring figure and ground. By measuring responses to figure and ground, we aimed to isolate FG-dependent signals, as a number of responses to a variety of natural patches would be expected to cancel out information other than figure and ground. We recorded activities from multiple neurons using array electrodes, so that the detailed CRF locations differed among neurons. Compared with a single electrode, array electrodes have the advantage of simultaneously recording from a population of neurons whose CRFs are distributed around an FG border. A number of natural patches, filled patches, and traditional square stimuli (Fig 1A & 1B; S1 Fig) were shown in sequence with a short duration.

In addition, we examined differential responses to pairs of each original patch and its variant patches. We generated variant patches corresponding to each of the original patches, with image characteristics similar to the original but different FG configurations. For natural patches, mirror images were created. For filled patches, translations and mirror images were created as shown in Fig 1C, which depicts the relation between the CRF center and the configuration of stimulus variants. In this representative case, the CRF is on the figure in the original but on the ground in the translation and mirror patches. It should be noted that the original and the variant share similar image properties, but the responses to the original and the variant are found to be responses to figure and ground, respectively. If the CRF center is close to the center of the original image, translation will likely allow us to obtain a neuron’s response to both figure and ground. In contrast, if the CRF center is off the center, mirror images are more likely to obtain responses to both. The translation variants share image properties with the original, including contour curvatures and their orientations, while the mirror variants share curvature but not orientation properties with the original. Both variants share Gestalt factors with the original, including convexity, closure, symmetry, similarity, and proximity.

### Distribution of CRF center and size

**Fig 3:**
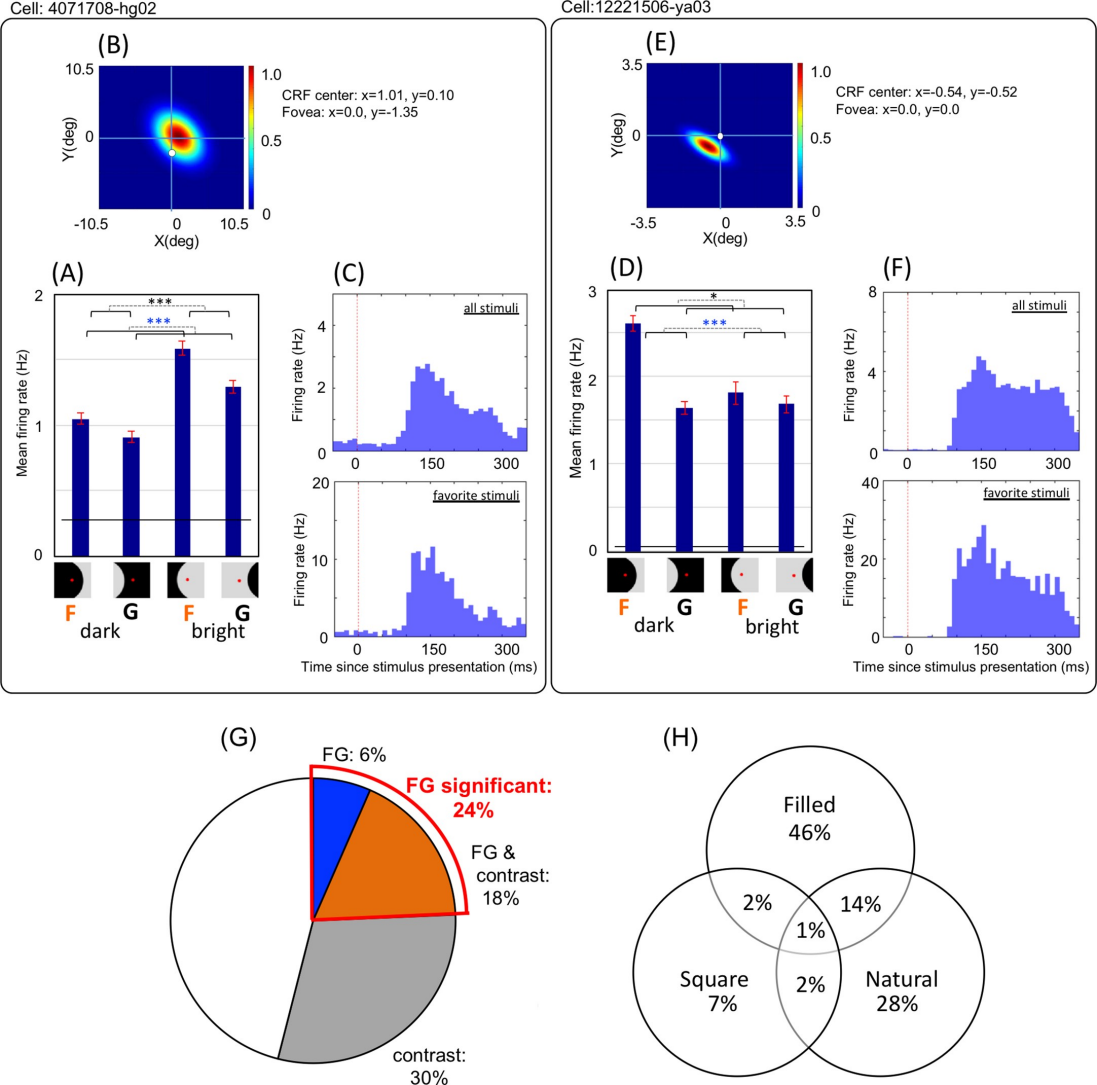
FG and contrast firing modulation of single neurons. A–F: Mean firing responses of two example neurons (each one is enclosed by a black rectangle) that showed significant FG modulation. A & D: Neural responses are sorted into four stimulus conditions according to the configuration of the stimulus patch and the neuron’s CRF center position indicated by the icons below. For example, the left-most bar indicates the mean spike rate across responses to dark figures. The level of the black bar indicates baseline firing rates, and the error bars indicate the standard error of the mean (SEM). Black and blue asterisks indicate the significance level (*: p < 0.05, ***: p < 0.001) of FG and contrast, respectively, computed from two-way ANOVA (refer “Data Analysis” section for details). B&E: The color map shows the spatial relationship among the stimulus extent (the outer square matches with that of patches in Fig 2), the CRF extent (shown in color with its center in dark red [1.0]), and the center of the fovea (white circle). In the case of the neuron on the right (E), the center of the stimulus (the center of the outer square) and the center of the fovea (the white circle) were located close to each other while the center of the CRF (the center of the colored ellipse) was slightly separated from them in the left-bottom direction. C&F: The histogram on the top is the peristimulus time histogram (PSTH) for all stimuli. The histogram at the bottom is the PSTH for the favorite stimuli. G: The percentage of neurons showing significant FG and contrast modulations among all recorded patch-responsive neuronal populations (793 neurons). Twenty-four percent of the neurons showed FG significance, based on raw firing counts. H: The percentage of neurons that showed FG modulation for each stimulus type. The panel shows the results of FG-significant cells in the MP sessions (106 neurons) where all three stimulus types were presented. The numbers in circles show the percentage of significant neurons that were mutually exclusive across stimulus types and their combinations. CRF, classical receptive field.

We applied spike sorting to all recorded activities during the entire period of each recording session, identifying a total of 2,244 neurons, including neurons that showed spiking activity regardless of stimulus presentation. Based on the responses to square-wave gratings presented at different spatial positions (in a 5 × 5 grid) on the monitor screen, we estimated the center position and the extent of the CRF for each neuron. Among the 2,244 recorded neurons, 793 neurons responded to patches, and their estimated CRF centers were located inside patches. We defined these 793 neurons as patch-responsive neurons, and used for the analysis (see “Data analysis” section for details). The eccentricity of the CRF centers ranged between 0 and 10 degrees, and the mean diameter of the CRF extent was 1.8 degrees. The estimated positions of the CRF centers and the frequency of their extent for an example single recording session are shown in Fig 2. The CRF sizes were in the range of those reported previously in V4 [31]. The spatial relations among the CRF center, fovea, and stimulus patch are shown for two example neurons in Fig 3B and 3E. As the locations of the CRF centers differed across the recorded neurons, we labeled each patch for each neuron based on the relationship between the image content of the patch and the center position of the CRF. For example, if a figure region fell onto the CRF center of the neuron, we assigned the label “figure” to the combination of this patch and neuron, and classified this patch for this neuron as a “figure patch.”

### Single-cell responses to natural and filled patches

We examined whether individual neurons exhibited FG modulation by comparing mean firing responses to figure and ground. Fig 3A–3F shows the responses of two example neurons exhibiting significant modulation to FG information. We focused on the population behavior of the neurons that showed FG-dependent responses, rather than the properties of single neurons. Thus, we analyzed the modulation across all stimuli, many of which did not evoke responses in individual neurons. Our rationale was that this evaluation reflected what actually happens in FG segregation when perceiving everyday natural scenes in which the optimal stimuli for a neuron rarely come into sight. The FG modulations and peristimulus time histograms (PSTHs; Fig 3C & 3F) included the responses to all stimuli, rather than the optimal stimuli. For comparison with other studies, we also examined the responses to “favorite stimuli”. We defined it as favorite stimuli if the response to the patch was greater than half of the peak response across all trials of the neuron. PSTHs across the responses to favorite stimuli are also shown in Fig 3C and 3F, showing strong activities that are consistent with previous studies in V4 [3, 7, 9].

We examined 793 neurons (416 and 377 in mirror-pair (MP) and translation-pair (TP) sessions, respectively) that showed significantly different responses during the presentation of stimulus patches compared with responses during the presentation of uniform grey (defined as patch responsiveness, see “Data analysis” section for details). As a population, 24.3% (193/793) of patch-responsive (p < 0.05, t-test) neurons showed significant (p < 0.05, ANOVA; see “Data analysis” section for details) FG modulation (Fig 3G). The mean modulation ratios (the mean difference in response between the preferred and non-preferred configurations divided by the overall mean) were 0.326 and 0.054 across FG-significant and non-significant cells, respectively. The significance ratio of 24.3% was smaller than those reported in previous studies [7, 9]. The result was expected, because previous studies selected neurons based on responsiveness to the specific stimuli presented, whereas we did not select neurons based on the responsiveness, but instead examined all patch-responsive neurons whose CRF centers were located on the stimuli. For comparison, we analyzed the recorded data based on responses to favorite stimuli (also refer to “Data analysis” section). The mean number of favorite stimuli across all neurons was 8.4, and the mean response rate was 36.7 Hz. The ratio of FG-significant cells increased to 43.9%, which appears consistent with previous studies. These results indicate that even when the analysis included all stimuli, regardless of how much neural activity they evoked, approximately one fourth of the neurons exhibited FG significance. In addition to FG-significant cells that preferred figure on the CRF, we observed a small number of FG-significant cells that preferred ground (24% = 46/193). From the viewpoint of neural representation, a combination of figure- and ground-preferred cells is likely to provide effective representation of FG organization.

Although we used the criteria of whether CRF center is on the figure or not, it is currently unclear whether FG labeling based on the spatial position of the CRF center reflects the nature of figures and grounds. The extent to which figural and ground regions fell on the CRF could affect the FG modulation of the neurons. We therefore examined an alternative measure in the analysis of FG modulation. Here, the labels “figure” and “ground” were defined based on the spatial extent of the overlap between a figure region and the CRF. If the extent of the CRF that overlapped with the figural region was larger than the extent overlapping with the ground region, the patch was labeled “figure” for this cell. Among the patch-responsive neurons, 24.0% (190/793) were classified as FG-significant using this labeling. This ratio was very similar to that classified by labeling based on the CRF center (24.3% = 193/793). The number of cells that were classified as FG-significant by the both methods was 141 (74% = 141/190). The detailed results are shown in S3 Fig. The similarity between the results computed from the two methods is likely to be related to a natural geometric tendency; when a region is divided into two areas, the center would most often belong to the larger area. In cases where the CRF is divided by a contour into one region with the center and one without, the central region would often have a larger extent than the peripheral region. The simplest scenario is a case in which the contour is straight (i.e., the central region is always larger). There could be exceptions in which the central region is extremely convex or thin, however, the number of such cases was small in the present stimuli.

We also compared FG modulation across natural patches, filled patches, and square images. The number of FG-significant cells was greater for filled and natural patches (46% and 28%, respectively), and smallest for square patches (7%), as shown in Fig 3H. The results revealed that 19% of the neurons showed significance for more than two types of stimulus sets. V2 neurons with border-ownership selectivity exhibited a range of consistency across a variety of stimuli (11–41%), including the consistency between squares and natural patches (around 35%) [15, 32]. The low percentage (19%) of inter-stimulus-type consistency for FG-significant cells indicates that individual neurons are not capable of FG discrimination for a wide variety of stimuli, suggesting that population coding underlies the representation of FG. The overlap between filled and natural patches was greater than the overlap between other combinations, potentially suggesting similar representations of the surface shapes defined by uniform grey in the filled patches and those defined by natural textures including shading in the natural patches. Although the number of FG-significant cells was greater for filled patches compared to natural patches with the criteria that the CRF center is on figure (46% and 28%, respectively), these ratios are similar to the criteria of the overlap between the CRF extent and figure (40% and 36%, respectively). Neural presentation of filled and natural patches would need to be investigated further.

We also examined FG modulation based on the difference between firing counts corresponding to either paired-translation or mirror images. Note that the analyses described so far in this section were calculated based on raw firing counts, meaning that the responses to single patches were summarized. The subtraction of responses to a translated patch from responses to the original patch would be expected to compensate for the activity originating from neuronal preferences with certain spatial invariance. For example, the neural activity originating from curvature selectivity in V4 neurons [33] would be expected to be compensated. With the subtraction in TPs, the percentage of FG-significant cells was 25% (p < 0.05, ANOVA, see “Data analysis” section for details). The subtraction in MPs compensates for the effect of contour features, except orientation. For example, the magnitudes of convexity, closure, symmetry, similarity, and proximity are identical within each pair, but their orientations are reversed. With the subtraction in MPs, the percentage of FG-significant cells was 23% (p < 0.05, ANOVA). The differential responses in the TPs and MPs yielded similar numbers of FG-significant cells compared to unpaired responses. Further analyses on pairing are provided in the following sections.

### Single cell-based figure-ground classification

**Fig 4:**
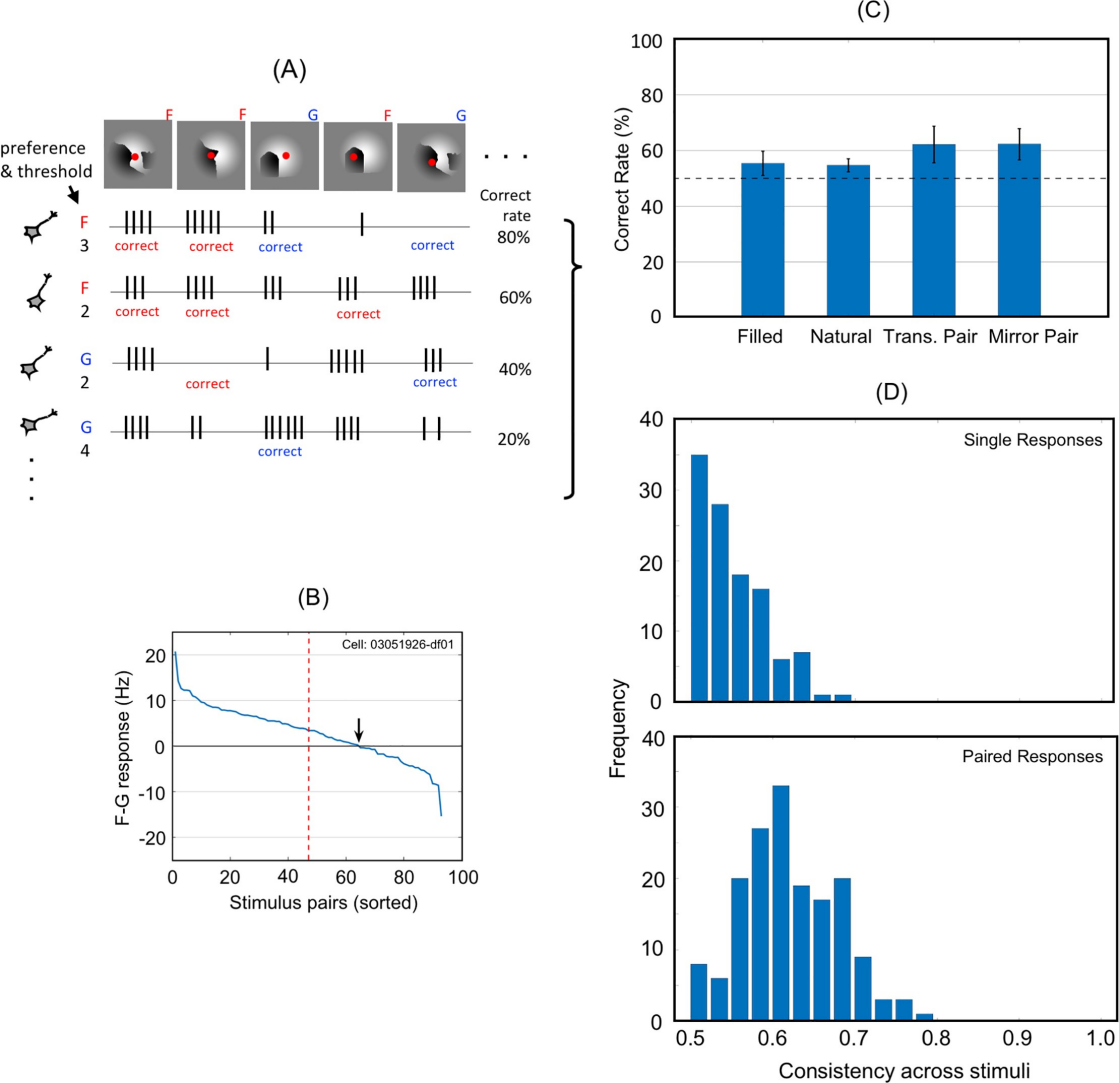
FG classification according to firing rates of single neurons. A: A schematic illustration showing the method to derive correct rates for single neurons from raw firing counts. This illustration shows four example neurons responding to five stimuli. The FG preference of a neuron was determined by the comparison between mean firing rates for figure and ground stimuli. A response to a stimulus is classified as *correct* if the response was greater than the threshold of the neuron when a preferred stimulus (the CRF center falls onto figure or ground) was presented. Similarly, a response is classified as correct if the response was smaller when a non-preferred stimulus was presented. The FG preference and threshold of the example neurons are indicated on the left side of the panel; F and G indicate the preference to figure and ground, respectively, and the numbers indicate the threshold in spike count. The spike trains below each stimulus illustrate the numbers of spikes as a response of the neurons to this stimulus. For example, the neuron at the top, which has preference to figure and threshold is three spikes/stimulus, showed four spikes to the left-most stimulus. This stimulus was preferred stimulus because the CRF center of this neuron (indicated by a red dot in the stimulus) fell onto figure (a white region in this stimulus). As the four spikes are greater than the threshold, this response is classified as correct. In this illustration, a total of five stimuli were presented, and the number of the correct responses of this neuron was four, so the correct rate of this neuron is 80%. B: The difference between the spike counts of figure and ground conditions for individual MPs in descending order. Data from an example neuron. Ninety-three pairs were usable for this neuron, where a figure region of one patch and a ground region of the mirrored patch fell onto the CRF center. The intersection of the curve with the abscissa (indicated by a black arrow) is shifted to the right of the center (a red dotted line) because the majority of patches produced stronger responses to figure than to ground. C: Mean correct rates across neuronal populations (193 FG-significant neurons). “Filled” and “Natural” indicate the stimulus types; rates were calculated from raw spiking activity. The correct rates for the differential responses were calculated from the responses to filled patches. Error bars indicate the standard error of the mean (SEM). D: The distributions of the correct rates (response consistency across the stimuli) were computed from single responses (top panel) and paired responses (bottom). Individual neurons barely discriminated FG across many stimuli.

To assess whether individual neurons are capable of FG discrimination in a variety of patches taken from natural images, we evaluated the consistency of FG signals across patches from single-neuron firing rates, as illustrated in Fig 4A (see “Data analysis” section for details on the method). First, to illustrate the variability of FG signal across stimuli, we plotted F − G responses of the individual patch-pairs for an example neuron (Fig 4B). The F − G response was defined as the difference between the response to the preferred region (figure or ground) and that to the non-preferred region of a mirror image pair. To achieve this, we selected usable patches in which a figure region fell onto the CRF center in one patch and a ground region in the mirrored patch. Fig 4B shows the computed F − G responses for the selected pairs (93) in descending order. The intersection of the curve with the abscissa is shifted to the right of the center because the majority of the patches evoked a stronger response to figure than to ground. The correct rate of this neuron was 68% (63/93). The results of the ANOVA showed that this neuron was FG significant (p < 0.001, F = 52.7), which was equivalent to the zero-crossing being significantly deviated from the center. It should be noted that the results of the ANOVA only revealed the significance of the shift, but it did not reveal the magnitude of deviation from the center. Rather, the degree of deviation is provided by the correct rate.

We computed mean correct rates across a population of neurons. For the analysis of responses to individual patches, the response was considered correct if a “preferred” (figure or ground) patch evoked a stronger response than a threshold. The threshold was determined for each neuron by using SVM so that the greatest correct rate was obtained. Although a thorough search could be used for the determination of the threshold, SVM was advantageous in generality and coherence with the population analysis (see “Methods and Materials” section for details). It should be noted that the method for plotting F − G responses is not applicable to responses to individual patches because it requires responses to stimulus pairs. The computed mean correct rates across FG-significant neurons were 55.4% and 54.8% for filled patches and natural patches, respectively (Fig 4C). With the differential firing counts for translation and mirror image pairs, the correct rates were slightly higher (62.4% and 62.2%, respectively; Fig 4C). These results indicate that the discrimination power of individual neurons is limited such that a single neuron is not capable of correctly discriminating a variety of stimuli. These neurons showed significant FG discrimination, but the correct rate was approximately 55–62%. This result could be clearly illustrated in the rank order of F − G signals; the deviation of zero-crossing from the center was significant, but only 5–12%. Although the FG modulation of these neurons was significant, the modulation appeared to be less rigid and robust than that previously reported in V4 neurons [3, 4]. It should be noted that our analysis included all neurons as long as patches fell on the CRF and responsive to patches. It is plausible that their optimal stimulus, even the preferred orientation, was different from the stimuli presented. As expected, we observed the lower responsiveness and modulation in our analysis compared with that reported in previous studies in which neurons were selected based on the selectivity to the presented stimuli. We consider that our method reproduced a naturalistic condition similar to viewing everyday scenes.

The distribution of correct rates for the neuronal population is shown in Fig 4D. The distribution was narrow around the mean, indicating that most neurons shared a lack of capability for FG discrimination across many patches. It could be argued that the low mean correct rate could originate from a combination of a small number of FG significant cells that are capable of correctly discriminating a variety of patches and a large number of FG cells with low correct rates. We observed no FG-significant cells showing high consistency, supporting the notion of population coding for FG discrimination in natural image patches. The distribution computed from paired responses revealed a similar distribution to that of single responses but with higher correct rates. The mean discrimination accuracy was similar but slightly smaller than that reported in a previous study of border ownership-selective cells [15]. In the distribution of paired responses, a small number of cells exhibited a high level of consistency (0.8) across most stimuli. The pairing may have canceled out other stimulus attributes and revealed the power of FG discrimination.

### Modulation latency

**Fig 5:**
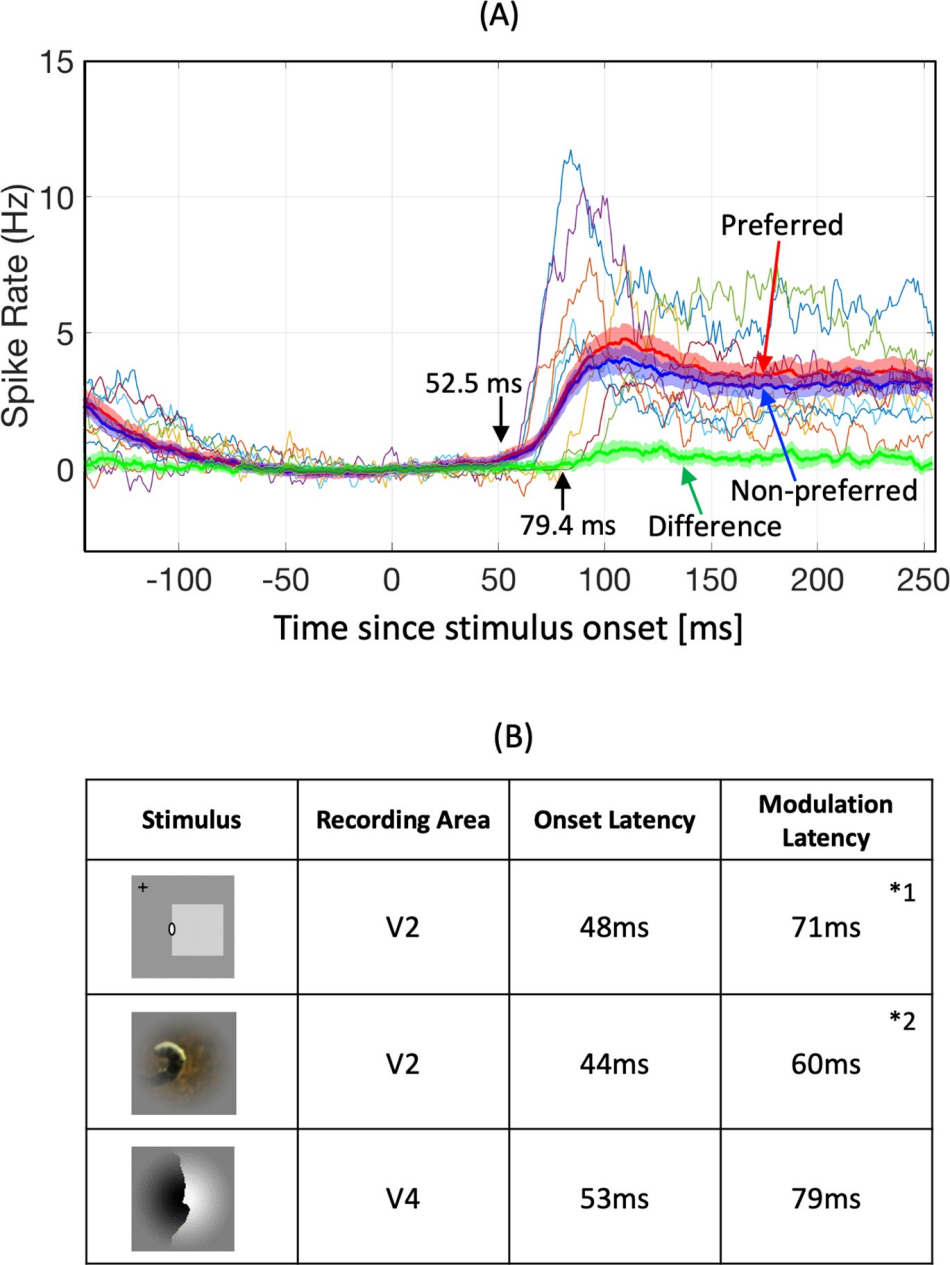
Time course of FG modulation (see “Data analysis” section for details). A: Thin colored lines indicate the example time course of the spike rate across all filled patches presented for 10 example neurons. The mean spike rates across the neurons showing FG significance to filled patches (80 neurons) were calculated separately for the preferred and non-preferred configurations, as shown by the thick red and blue lines, respectively. The subtraction of the two traces as the time course of FG modulation is shown by the thick green line. Shaded error bars indicate SEM. B: Summary of the onset and modulation latencies. The latencies for the border ownership-selective cells reported in previous experiments are also shown for reference (*1: [29]Sugihara et al., 2011, *2: [15]Williford and von der Heydt, 2016).

To examine the neuronal dynamics of FG modulation, we computed response-onset latency and FG-modulation latency from the responses of FG-significant cells to filled patches recorded in MP sessions (87 cells). We excluded neurons showing suppressive responses during stimulus presentations (seven cells) from the latency estimation. The time courses of the mean responses across all neurons examined, calculated separately for the responses to preferred and non-preferred FG configurations, are shown in Fig 5A (red and blue thick lines). The difference between the mean responses to preferred and non-preferred configurations is also shown in the figure (thick green line). Onset and FG-modulation latencies of the mean responses across the neurons were 52.5 and 79.4 ms, respectively (computed from the summation and subtraction of the two traces, respectively; R^2^ values for the regression were 0.986 and 0.957, respectively). The delay between the response onset and the modulation onset was 26.9 ms, indicating that early FG representation occurred in less than 27 ms. This modulation latency is consistent with previous reports of border ownership modulation in V2 (Fig 5B) [15, 29]. It might be difficult to draw firm conclusions from the latencies alone regarding whether the FG modulation we reported here depended on bottom-up signals or top-down feedback from higher areas. However, this short modulation-latency provides crucial information for the understanding of computational mechanisms underlying FG determination. A more detailed discussion of the latency is provided in the Discussion section.

### Population-based figure-ground discrimination

Individual neurons with FG-dependent activity responded only to a subset of stimulus patches. These single neurons showed a low level of consistency in FG discrimination across all image patches. Thus, single neurons were not capable of correctly signaling figures and grounds across a variety of stimuli. We hypothesized that population coding enables the robust representation of FG across a wide variety of natural patches. We examined whether the activities of multiple neurons were capable of signaling figures and grounds with high consistency across all patches. Given that it is currently unclear how neural activities are integrated to enable FG signaling, we used SVM as an ideal integrator to group the responses of multiple neurons. Specifically, we trained SVM to classify figures and grounds from the firing activities of multiple V4 neurons. We expected that the machine learning algorithm would achieve a higher classification rate compared with single neurons if the firing activities of multiple neurons contained information necessary for FG discrimination. If this was the case, population coding of FG in V4 would be predicted. However, the mechanism by which the activities of multiple neurons are integrated *in vivo* remains unclear.

**Fig 6:**
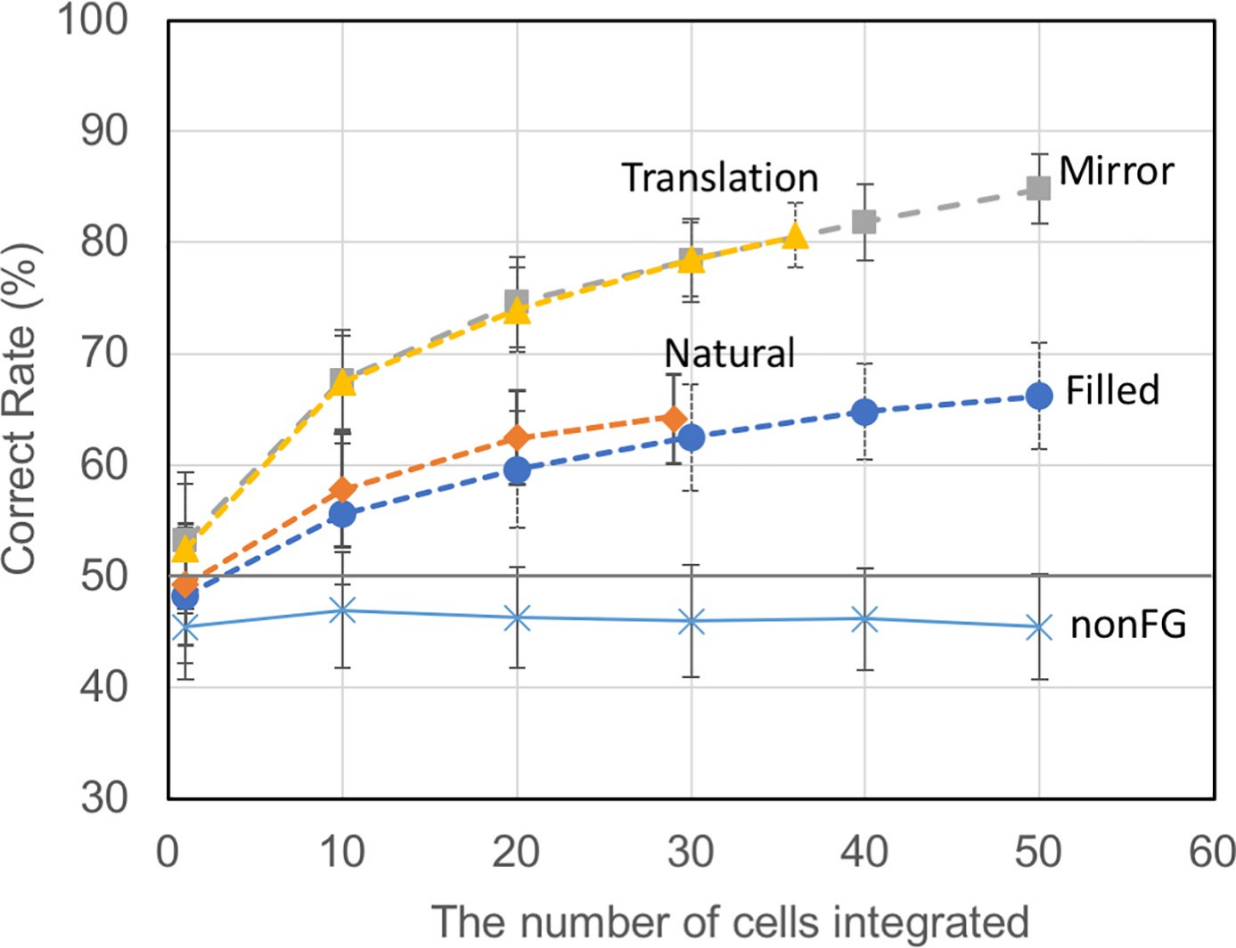
The computed correct rates of FG classification across stimuli as a function of the number of integrated neurons. The computed correct rates from the responses of neurons with FG significance for the filled patches (blue circles), natural patches (red diamonds), paired-mirror patches (grey squares), paired-translation patches (yellow triangles) and those of neurons without FG significance (blue crosses). The correct rate increases as the number of integrated neurons increases when FG-significant cells were pooled. The simulation was repeated 200 times for every condition with randomization. The graphs show the mean across the simulations, and the error bars show the standard deviation. Note that the correct rates for single cells (1 on the abscissa) are often below chance (50%) because of the cross validation used in the SVM computation (refer to “Support vector machine” section).

It is unclear how many neurons are integrated for FG discrimination and which neurons should be integrated, so we randomly sampled one to a few tens of FG-significant cells and computed the correct rates as a function of the number of neurons for integration. The spike counts of the sampled neurons in response to single stimuli were fed into SVM with the veridical FG-labels provided by human psychophysical experiments. Since the numbers of FG-significant cells recorded in single sessions were limited, we randomly selected FG-significant cells across all recording sessions. The computed correct rates for FG-significant cells with filled patches are shown in Fig 6 (blue circles). The correct rate increased as the number of neurons for integration increased: 55.6% and 66.2% for 10 and 50 neurons, respectively. The computed correct rates for natural patches showed the same tendency as those for filled patches (Fig 6 red diamonds). The correct rates for natural patches (57.8 and 64.2% for 10 and 29 neurons, respectively) were slightly higher than those seen for filled patches, but the difference was not statistically significant. The perception of figure and ground in natural images has been previously reported to fluctuate [11]. The veridical labels of figure and ground for patches were determined from human judgments; therefore, the labels exhibited limited consistency. The mean perceptual consistency in FG judgments across the patches was 0.73 (see S1 Appendix for details). If this perceptual consistency is considered as the basis of FG discrimination, the computed correct rates of around 65% would indicate a high level of agreement between perception and estimated neural performance.

Sampling of neurons across recording sessions was advantageous in analyzing a large number of neurons because the maximum number of FG significant cells recorded in a single session was 15. However, it could be possible that real-time interactions across nearby neurons affected the results. Sampling within a single session could also induce correlated noise in the simultaneous recording [34, 35]. The results from single and multiple sessions could be different. To rule out such effects, we performed the same analysis for neurons sampled within a single session. The correct rates for filled patches were estimated from the responses of ten neurons that were sampled randomly within a single session. With six sessions in which ten or more FG-significant cells were recorded, the estimated correct rate ranged between 55.8% and 69.5% (mean = 63.1%, SD = 5.6). The correct rate estimated from ten neurons sampled randomly across the six sessions was 64.0%, indicating marginal difference between the two sampling methods. We did not find evidence that sampling across sessions affected the results.

Neuronal populations without FG significance would be expected to exhibit chance rate or lower correct rates than those with FG significance. The results for the non-significant populations are shown in Fig 6 as blue crosses, indicating the correct rate close to the level of chance independent of the number of neurons integrated. Regarding a common concern in machine learning experiments, it could be argued that the power of SVM is extremely high and that it is thus capable of classifying neural activity for any criterion. For instance, if we label randomly spiking activities and run SVM, it could correctly classify the activity according to the label. If this were the case, the present results would not support the capability of neurons for FG discrimination. The chance rates obtained for non-FG-significant cells, however, rule out this possibility.

We trained SVM with the differential responses by using the same method. The computed correct rates for the paired responses were higher than those obtained for the unpaired responses, as shown in Fig 6. The MPs yielded rates of 67.6% and 84.8% for 10 and 50 neurons, respectively (grey squares), and the TPs yielded rates of 67.4% and 80.7% for 10 and 36 neurons, respectively (yellow triangles). The observation of higher correct rates for the paired responses than the raw responses was expected, because the paired responses showed greater correct rates in the single-cell analyses (Fig 4C). The pairing might have canceled out the responses attributable to other stimulus properties that were common between the original and paired stimuli, and clarified the difference between the responses to figure and ground. Although the paired responses would never be obtained in real-world situations, the analyses of paired responses were effective in unveiling the power of FG discrimination. The paired responses of the neural populations without FG significance produced correct rates close to chance rate (mean: 42.8–46.7%, SD: 2.8–5.3), as similar to the unpaired responses. These results indicate that FG-significant neuronal populations, which constituted around one fourth of patch responsive neurons in V4, contain information about FG segregation. The finding of higher correct rates achieved by population integration suggest that a few tens of neurons in V4 can represent FG information in a distributed manner.

## Discussion

In the hierarchy of the visual system, bottom-up and top-down signals, including local image features and object knowledge, are integrated to produce understanding of the scene. To understand the nature of FG information obscured in natural images as well as its neuronal representation, we investigated the potentials of neuronal activities to signal figures and grounds embedded in a variety of everyday natural scenes. We measured neuronal activity in response to local image patches and examined the modulation of activity related to figure and ground in the absence of global context. Approximately one fourth of the patch-responsive V4 neurons showed modulation of firing activity depending on the positional relation between the CRF center of the neuron and the figural region of the stimulus. However, most of the single neurons showed strong responses to a small number of patches, and weak or no responses to many patches. Thus, those neurons resulted in low levels of consistency in FG judgment across patches, suggesting that single neurons are capable of discriminating FG only for a limited portion of natural image patches. The relatively early latencies of FG modulation and their development over time after stimulus onset suggest that this modulation is likely to be substantially based on interactions with lower areas, and then refined over time. Our results are consistent with previous reports of FG modulation of V4 neurons in single cell recordings with artificial images [3, 4, 6]. Furthermore, we used SVM to analyze whether the activities of a small group of V4 neurons include information capable of FG discrimination across a wide variety of natural patches. The population activity of a few tens of FG-significant neurons exhibited high consistency across stimuli, up to around 85% (for 50 neurons), suggesting efficient distributed coding of FG information in V4. The results revealed that the power of single cells in FG discrimination across the natural image patches was weak but the integration of a few tens of cells was capable of achieving correct discrimination (up to 85%) among a variety of patches. The integration of responses from multiple neurons with different optimal stimulus is widely considered to enable the representation of an object or its attributes. We provided evidence for population coding of FG across a variety of natural patches. The present results revealed that the implicit information contained in local natural images can contribute to the representation of figure and ground. The findings of previous experiments with simplified stimuli should be investigated under more natural and realistic conditions in future studies to elucidate neuronal information processing in more detail.

Responses to natural image patches, compared with responses to filled patches, yielded a slightly better FG classification performance in multiple-cells (SVM) analyses, though the difference was not statistically significant. While the contours included in natural and filled patches are the same, the other content features, including texture, color, and shading, could improve neuronal FG discrimination. Ramenahalli *et al*. [14] reported that local spectral anisotropy between the figure and ground sides along a contour was capable of yielding a rate of correct FG assignment of almost 70%, using SVM. This result suggests that texture and shading include a fair amount of information that can be used for FG discrimination, although it is currently unclear how much information is used by V4 neurons. The neurons in V4 that are selective to texture, luminance/color gradient, and shading [36–38] could be expected to show joint selectivity for FG discrimination, and contribute to better discrimination performance. It has been reported that natural images are easier to discriminate than artificial images, regardless of attention or learning effects [39], and that natural image and video stimuli evoke more reliable and reproducible neural activity than simple stimuli [40]. The responses of V1 neuron pairs were found to be more strongly de-correlated with natural than artificial images, suggesting more efficient coding for natural images [41]. The visual system presumably evolved to be optimized for the interpretation of natural images, probably based on their statistical characteristics.

Natural images enabled us to examine a variety of shapes compared to artificial shapes such as squares and parameterized shapes, which are crucial for understanding the construction of object representation from everyday natural scenes. A notable difference between the present stimuli and the artificial stimuli is observed in perceptual consistency. Perceptual FG assignments, which parts appear figure and ground, in the artificial stimuli are straightforward so that the consistency across observers and repeats is close to perfect, whereas the FG assignment in the present stimuli taken from natural images varied across observers and repeats so that the consistency is lower. Our psychophysical experiments showed that the mean consistency in FG assignment was 0.77 and 0.69 for filled and natural patches, respectively. These perceptual consistencies would be considered as the ground truth in the analysis of neural data. Our population study with SVM showed up to around 70% in correct rate, which might be close to the highest possible correct rate that is given by the perceptual consistency. An analysis that bridges the perceptual consistency and neural data might be an interesting future work.

The stimulus set in the present study included several natural patches in which a large portion of an object was visible, enabling the object (e.g., a human face or a silo) to be recognized. Specifically, eight natural patches included the contextual information as indicated by # in S2 Fig. The perceptual consistency among the eight contextual stimuli was 0.78 which was 13% (= 0.78/0.69) greater than the consistency across all natural-patches. The mean neural correct-rate among these contextual stimuli was 0.59, which was 7% (= 0.59/0.55) greater than the mean correct rate across all natural-patches. Although we are not able to draw a conclusion on the correlation between the perceptual consistency and the neural correct-rate as the number of the contextual stimuli is small, it would be interesting to further investigate the correlation between the perception and neural responses. We identified eight contextual stimuli among natural patches but none among color-inverted patches and filled patches. The ratio of contextual stimuli among natural patches was 3.8% (8/210). Taking into account the 7% difference in neural correct-rate, we estimate that over all contribution of the context in the natural patches (0.27%) is negligible for our analyses. Since the contextual information is a crucial cue for FG determination, further analyses of such stimuli would be expected to provide useful information for understanding the neural mechanisms involved.

A study on border-ownership modulation for natural scenes in V2 reported that approximately half of the recorded V2 population showed border-ownership modulation in response to full-size natural images (full context), while fewer neurons showed modulation to natural patch images [15]. Specifically, response consistency to natural patch images was approximately the level of chance. Although the recording areas were different, it is useful to compare these results with the current findings. First, it should be noted that our natural patches were three times larger than the estimated CRFs, whereas the natural patches used by Williford and von der Heydt [15] were almost the same size as V2 CRFs. Second, the selection of stimuli differed. Our stimuli were intended to be as realistic as possible [13]. We included a wide range of convexity, closure, and width and shape of objects, and did not control the complexity of FG judgments, such as whether both figure and ground fell on the CRF. In contrast, Williford and von der Heydt selected stimuli manually, excluding patches with high convexity and thin objects as well as stimuli in which both sides of the object fell onto the CRF. Third, the sampling of neurons also differed. We recorded V4 neuronal activity using array electrodes and performed no pre-selection for the recording and analysis of each neuron. In contrast, Williford and von der Heydt used single electrodes and recorded from orientation-selective cells. Despite these differences, the two studies are consistent in reporting a substantial number of neurons with border-ownership- or FG-modulation in response to natural stimulus sets. Consistency between the two reports is also found in the reported latencies: border-ownership and FG modulation were observed at approximately 20 ms after the response onset, suggesting that modulation is a rapid process and not dependent on slow feedback signals from higher ventral areas. One might argue that FG-significant cells could be the grouping cells in a hypothetical model where the grouping cells in V4 feed crucial border-ownership information to V2 neurons [42]. The grouping cells should show short latencies, because they need to signal prior to the border-ownership modulation in V2 (60 to 71 ms). The observed modulation latency for FG-significant cells was 79.4 ms, which was slightly longer than the reported V2 border-ownership modulation latency, thus the FG-significant cells might not meet the requirement for the grouping cells. Based on this small latency difference and detailed methodological differences between laboratories, we might not be able to rule out the V4 grouping cell hypothesis. Further investigation will be necessary to clarify the circuit-level functional meaning of the observed FG modulation in V4.

In the present study, we observed a response onset of 52.5 ms and FG modulation onset of 79.4 ms. Interestingly, a study on shape selectivity in V4 reported that the shape modulation started around 50 ms and reached a peak at around 100 ms [43]. These observations indicated that FG processing began while shape selectivity was still developing. Although the interpretation of these observations might not be straightforward, the recurrence of signals might be expected to pass through V4 for the computation of figure and ground. There are several possibilities regarding the source of FG modulation observed in V4 in addition to the feedforward signals from V2. Anatomical studies reported intensive convergence in the connection from V4 to TEO, and extensive feedback from TEO to V4 [44]. However, the response latency of TEO neurons that showed early latencies was 93 ms on average [45]. Thus, the feedback from TEO might not contribute to the FG modulation latency. Feedback through V2 (V4->V2->V4) is another possible scenario, as mentioned above. Lateral interaction across V4 neurons is also possible because the intrinsic connection in V4 is more extensive compared with the lower visual areas [46]. To clarify the source of the observed FG modulation, other types of recording, such as layer-specific recording, might be necessary, which will be a possible future experiment.

In discussing the roles of top-down vs. bottom-up processing, the possibility that some effects originated from the conditions of animal preparation, including anesthesia or paralyzation, should be considered. For instance, differential effects of isoflurane on bottom-up and top-down connectivity have been reported in the rodent auditory cortex [47]. In the present study, single neurons in V4 of analgesized monkeys exhibited weak FG modulation throughout the stimulus presentation, with a modulation latency of 79 ms. If animals were awake and engaged in a visual task, it might be expected that neurons would show strong FG related activity, especially in the latter part of the neural activity where feedback from higher areas and recurrent signals through lower regions are effective. A previous study reported the suppression of FG related activity in V1 of anesthetized monkeys [48], while we found significant FG related neural activity in V4 of analgesized monkeys. One possible cause of the contrasting results is the difference in anesthesia. We used fentanyl citrate, which alleviates pain without causing loss of consciousness, whereas the previous study used isoflurane which causes loss of consciousness. Another possible cause of the contrasting results is the difference in recording area. We recorded neural activity from area V4, whereas the previous study recorded from V1. It might be possible that suppression of feedback from V4 to V1 by anesthesia resulted in the loss of FG related activity in V1. If an essential role of the analgesia was suppression of top-down connections, it might be suggested that V4 neurons are capable of determining local FG, at least in part, from feed-forward signals. In any case, it is important to study neural activity of V1 and V4 in a same awake condition in a future study.

The mean correct rates of single cells computed from the differential firing counts for translation and mirror image pairs (62.4% and 62.2%, respectively) were slightly higher than those computed from single patches (55.4%). The distribution of the correct rates computed from the paired responses appeared biased toward a higher range, compared to the distribution computed from the single responses (Fig 4D). Our SVM analysis also showed that paired inputs induced higher FG discrimination performance than un-paired inputs (Fig 6). We estimated the correct rates based on the differential activity in pairs, with the aim of cancelling out image characteristics other than FG. The analyses based on differential activity in pairs are common across electrophysiology; for example, subtraction in mirror pairs has been utilized in testing border-ownership selectivity [e.g., [5]. The subtraction in translation pairs compensates the effect of contour features. The magnitudes of convexity, closure, symmetry, similarity, proximity, and their orientations, as well as curvature and contour shape, are identical within each pair, and thus the subtraction tends to cancel out them as long as they are spatially invariant. The subtraction in mirror pairs compensates the effect of the contour features except orientation.

The cancellation of image characteristics was likely crucial for the high performance obtained from the differential activity. However, it could be argued that the high performance reflects some feature of neurons. Computational studies on border ownership selectivity in V2 reported that paired units with respect to the side of the figure were able to reproduce the phenomena observed in border ownership-selective cells [42, 49]. A similar pairing mechanism with respect to figure/ground regions would be expected to reproduce better accuracy with paired inputs, although the mechanism needed to work effectively with the asynchronous presentation of pairs. It has been reported that the representation of stimulus shape depended on local symmetry [50]. This may be related to the dominance of the detector for symmetrical shape over that for asymmetrical shape in the visual system. Psychophysical studies have shown that symmetry processing is a pre-attentive visual process that forms an integral part of perceptual organization [51, 52]. Although the mechanisms underlying symmetry detection are still unclear, it is possible that the system positively utilizes mirror images in natural scene understanding. Translation pairs could be considered similarly since the region between the translation pairs likely appeared figure because of parallelism. Investigations on the high consistency of paired responses might provide insight into the cortical mechanisms underlying FG coding and shape representation.

The results of our SVM analyses showed intermediate correct rates, such as 66.2% for unpaired responses. However, absolute values would not provide essential information, because a better classifier could achieve a higher correct rate. The present results are however qualitatively meaningful, as a higher correct rate indicates a greater capability for FG discrimination. The results of our SVM analysis suggest that FG information is coded in a distributed manner. The SVM analysis with paired responses achieved a performance of around 85% correct for 50 neurons. These high correct rates suggest that a few tens of neurons, or less, include the information necessary for FG segregation in natural images. Given that dimensionality represents the necessary number of cells for a representation, the dimensionality of the FG representation in V4 appears to be approximately 50 or less. Recent physiological studies have reported that the approximate dimensions for the representation of face identity and objects in IT are 50 and 100, respectively [53, 54], consistent with the approximately 50 dimensions in V4 for FG segregation in natural images.

Elucidating how the population signals represent figural regions and how they are read out by later neural stages is important for advancing the understanding of the essence of visual processing. FG segregation involves the integration of visual features belonging to each object. An image projected onto the retina is decomposed into visual features, and they are integrated separately for each object to form proto-objects [55, 56]. Proto-objects can be considered as early- or intermediate-level representations of an object, which can be constructed based on perceptual organization [57]. The cortical area V4 is a suitable candidate for the basis of proto-object, and the neuronal activity reported here appears to be relevant to this type of processing. Although how proto-objects are represented in the cortex has not been clarified, computational studies have proposed several models. Craft *et al*. [42] proposed a grouping-cell model that directs a certain circular region to be integrated. It may be valuable to consider different populations of neurons for the detection of visual features and the instruction of integration. Martin et al. [58] proposed that temporal coding could be based on the synchronization of activity. Sakai and Nishimura [59] suggested that the anisotropy in surrounding effects, or that in grouping of early visual features, can provide the direction of a figure. Super et al. [19] suggested that multiple feature maps can be constructed through feedforward mechanisms. It is assumed that the proto-object is then read out by neurons in higher cortical areas to form the percept of the object. The readout may rely on similar mechanisms to the formation of the proto-object. A single neuron or a population of neurons could provide signals that determine which regions should be grouped [60]. Alternatively, temporal mechanisms such as synchronization could also direct the grouping process. Attention appears to play an important role in the integration involved in forming the percept of an object [61]. Investigation on the representation of figural regions together with the corresponding neuronal read out could further advance current understandings of the recognition of visual scenes. We are making the data from our experiments available [62] in order to enable comparison of model simulations with neural data.

Previous physiological studies have reported that neurons in V4 are selective for multiple stimulus features, including curvature [33], texture [38], and 3D orientation [63]. These are all key features for the judgment of FG segregation in natural scenes. It may be valuable to investigate whether these features show simple selectivity to multiple stimulus features or share properties with those observed in mixed selectivity [37, 64]. Further investigations of such neuronal features in relation to FG modulation will be needed to elucidate the cortical mechanisms underlying natural scene understanding in more depth.

## Supporting information

S1 Fig Linear (a single shank) and comb (8 shanks) probes (refer to “Recording of neural activity” section) were used in 4 and 20 recording sessions, respectively, and recorded 90 and 2063 isolated single units, respectively. (A): The histogram of the number of units recorded by a linear (orange/bright bars) and comb (blue/dark bars) probes. (B): The histogram of the mean firing rate across the units. The distributions of the linear and comb probes overlap substantially. We did not find evidence that the two types of probes show substantial difference in recording.
(TIF)

S2 Fig The set of natural and filled patches used in the experiment, with variants including mirrored, translated, and contrast-reversed patches (not shown here). Black and white regions in filled patches correspond to figure and ground, respectively, which were determined based on the human psychophysical experiment described in S1 Appendix.
(TIF)

S3 Fig FG and contrast modulation of single cells with FG labels based on the overlap of the CRF extent and a figure region. A: Percentage of neurons showing significant FG and contrast modulations among all recorded patch-responsive neuronal populations (793 neurons). A total of 24% (190/793) of the neurons showed FG significance, which is the same ratio computed with FG label based on the location of the CRF center. B: Percentage of neurons showing FG modulation for each stimulus type. The panel shows the results of FG-significant cells in MT sessions (106 neurons) where all three types of stimuli were presented. The conventions are the same as in Fig 3.
(TIFF)

S1 Appendix (DOC)
