# Supplementary figures and images for: Population coding of figure and ground in natural image patches by V4 neurons
Source: PLoS One. 2020 Jun 26;15(6):e0235128. doi: 10.1371/journal.pone.0235128 (PMC7319327; doi:10.1371/journal.pone.0235128)

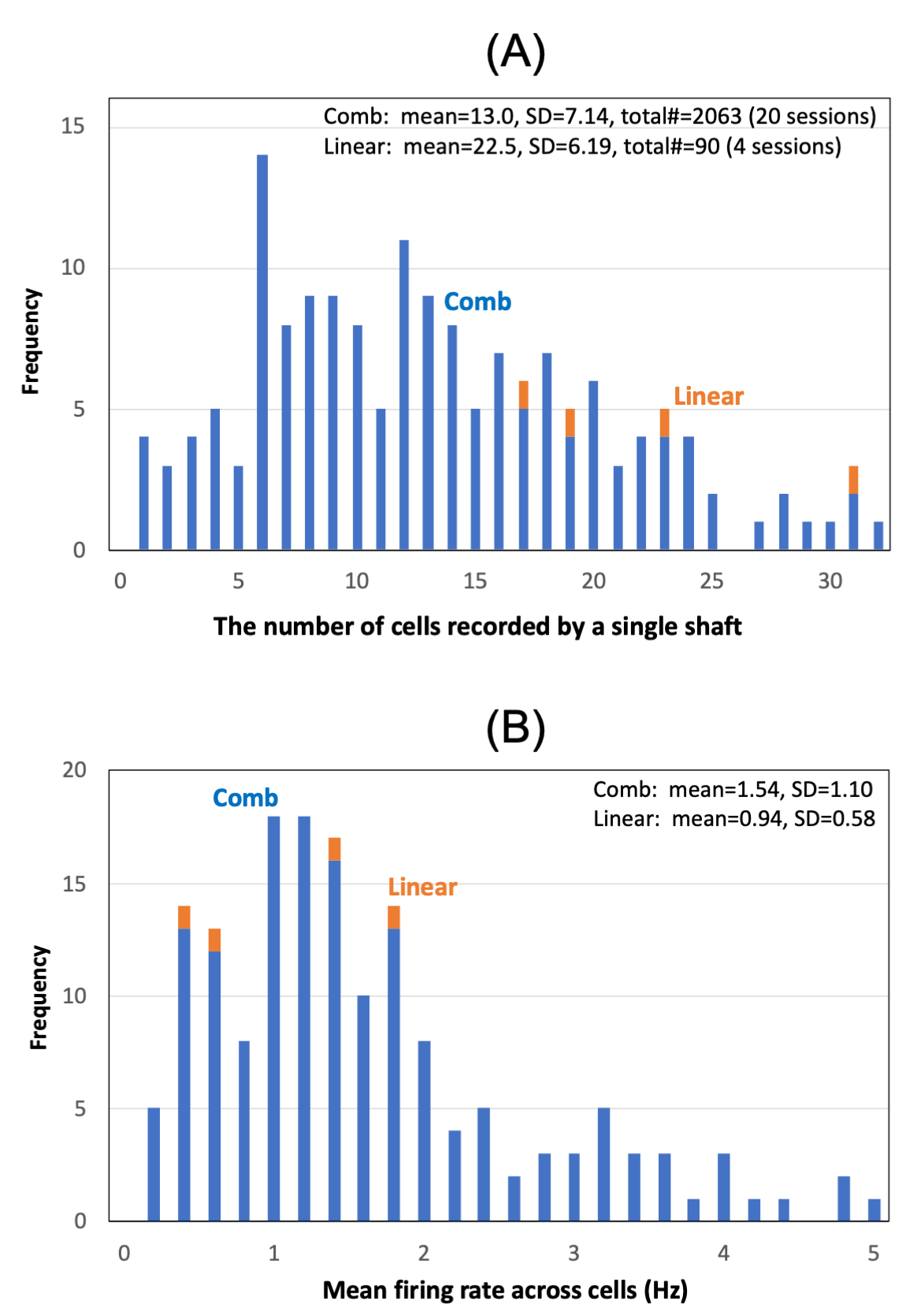

Supplement: S1 Fig — (A): The histogram of the number of units recorded by a linear (orange/bright bars) and comb (blue/dark bars) probes. (B): The histogram of the mean firing rate across the units. The distributions of the linear and comb probes overlap substantially. We did not find evidence that the two types of probes show substantial difference in recording. (TIF) [file pone.0235128.s001.tif]

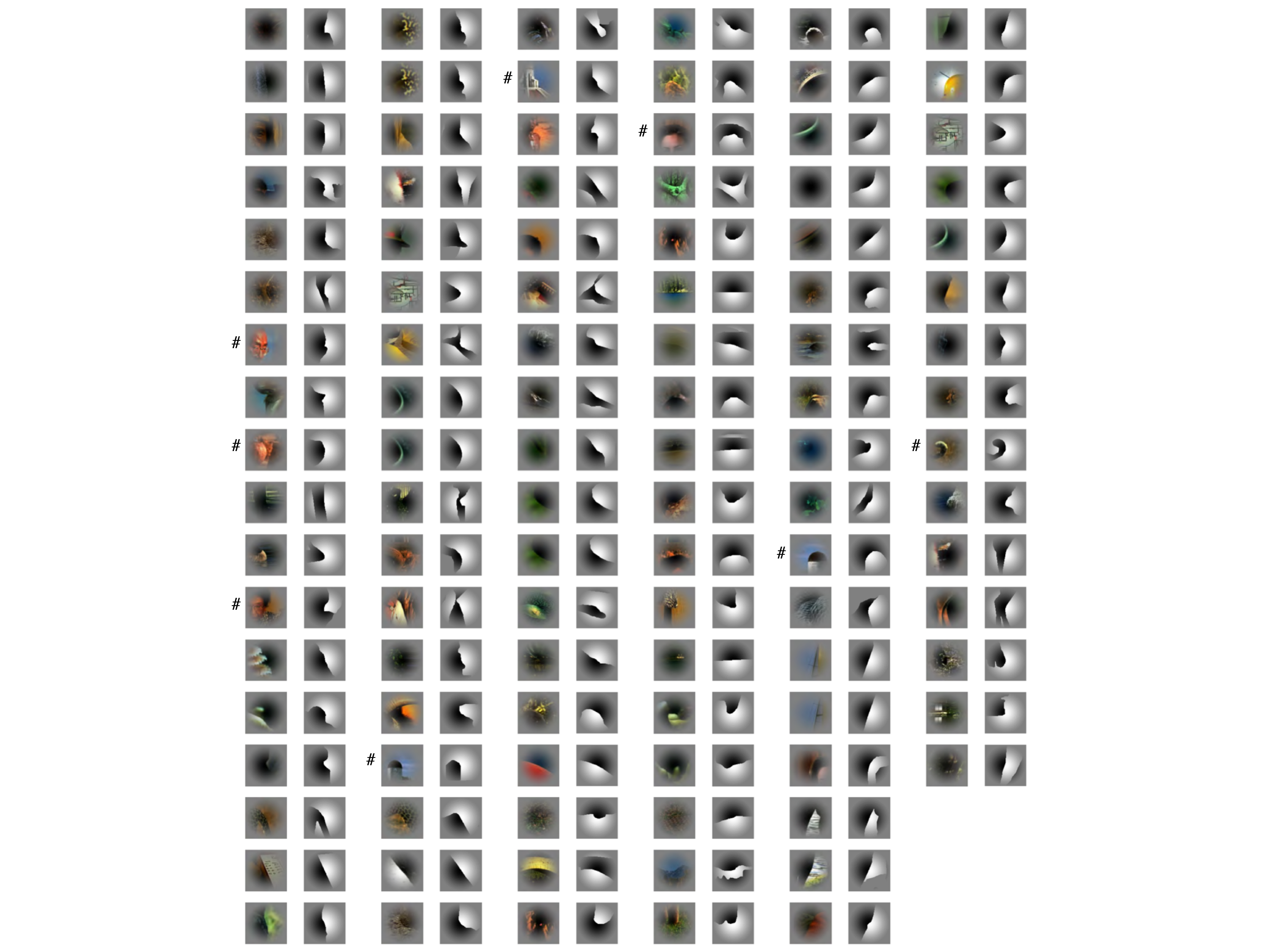

Supplement: S2 Fig — Black and white regions in filled patches correspond to figure and ground, respectively, which were determined based on the human psychophysical experiment described in S1 Appendix. (TIF) [file pone.0235128.s002.tif]

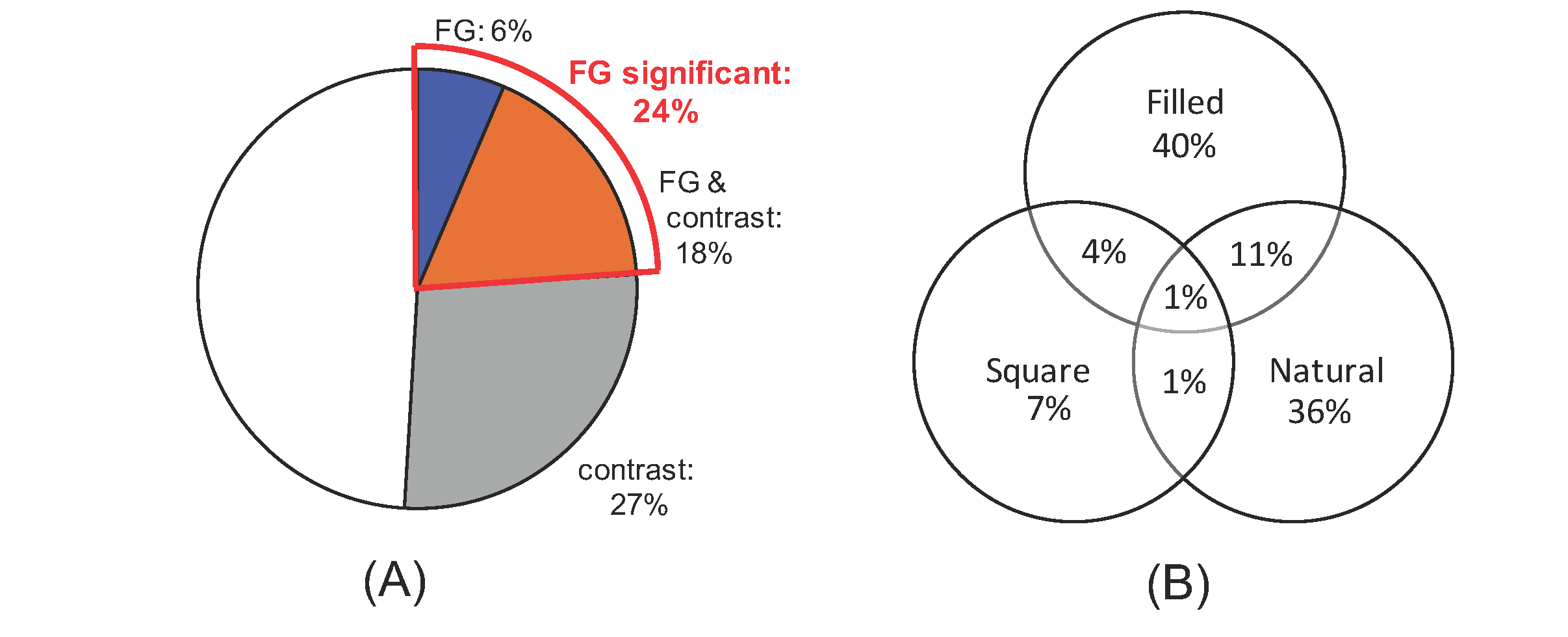

Supplement: S3 Fig — A: Percentage of neurons showing significant FG and contrast modulations among all recorded patch-responsive neuronal populations (793 neurons). A total of 24% (190/793) of the neurons showed FG significance, which is the same ratio computed with FG label based on the location of the CRF center. B: Percentage of neurons showing FG modulation for each stimulus type. The panel shows the results of FG-significant cells in MT sessions (106 neurons) where all three types of stimuli were presented. The conventions are the same as in Fig 3. (TIFF) [file pone.0235128.s003.tiff]
