# Supplementary material for: Population coding of figure and ground in natural image patches by V4 neurons
Source: PLoS One. 2020 Jun 26;15(6):e0235128. doi: 10.1371/journal.pone.0235128 (PMC7319327; doi:10.1371/journal.pone.0235128)
Supplement: S1 Appendix — (DOC) [file pone.0235128.s004.doc]

**Appendix A**

To determine the veridical label of figure and ground to natural and filled patches, we performed a human psychophysical experiment. This appendix briefly describes the procedure and result of the experiment. For natural patches, we tested 210 patches (the original and mirror for 105 types of patch) with three repeats. Eight participants (age: 21-40; 2 females and 6 males) performed the experiment. All participants had normal or corrected-to-normal vision. The results were pooled for each patch-type (105), and the consistencies were computed for each patch-type. The consistency was defined as the difference between the number of more chosen direction and that of the other direction, divided by the sum of the two, thus it ranges between 0 and 1. For filled patches, we tested 420 patches (the original and mirror, as well as their contrast reversals) with three repeats. Seven out of eight participants who carried out the experiment with natural patches performed the experiment. The consistency was computed in the same way as for the natural patches. The procedure was identical to [31], except for the test patches. The test patches were rotated up to 45 degree so that the tangent of a contour that passes through the patch center was either vertical or horizontal. Patches with the vertical contour at the center and those with the horizontal contour were shown in distinct sessions. A single patch was presented for 500 ms. The participants were instructed to report the direction of the figure from the center of a patch using two-alternative forced choice paradigm (up or down for the horizontal contours; right and left for the vertical). The mean measured consistencies across patches were 0.69 and 0.77 for natural and filled patches, respectively (SEM=0.11 and 0.14, respectively). Figure-ground labels used in the analyses of the present study were based on the result of this experiment. The region in the direction of perceived figure from the patch center was labeled as figure and the other regions were labeled ground. The region was defined as the extent surrounded by human-marked contours (from Berkeley Segmentation Dataset) and the perimeter of the patch. Note that the stimuli were intended to include patches with a wide range of consistency as observed here because we focused on the behavior of neurons in response to natural patches and shapes.

Written informed consent was obtained from all participants prior to the experiment. All experiments were performed in accordance with the guidelines of the Japanese Psychological Association and the Code of Ethics of the World Medical Association (Declaration of Helsinki), and they were approved by the Research Ethics Committee of the Faculty of Engineering, Information, and Systems at the University of Tsukuba (certificate no: 2014-R52-2).
